# Incidence of bone metastases and skeletal-related events in breast cancer patients: A population-based cohort study in Denmark

**DOI:** 10.1186/1471-2407-11-29

**Published:** 2011-01-24

**Authors:** Annette Ø Jensen, Jacob B Jacobsen, Mette Nørgaard, Mellissa Yong, Jon P Fryzek, Henrik T Sørensen

**Affiliations:** 1 Department of Clinical Epidemiology, Aarhus University Hospital, Ole Worms Alle 1150, DK-8000 Århus C., Denmark; 2 Center for Observational Research, Amgen Inc., One Amgen Center Drive, MS 24-2-A, Thousands Oaks CA 91320, USA

## Abstract

**Background:**

Breast cancer (BrCa) is the most commonly diagnosed cancer among women in the industrialized world. More than half of women presenting with metastatic BrCa develop bone metastases. Bone metastases increase the risk of skeletal-related events (SREs), defined as pathological fractures, spinal cord compression, bone pain requiring palliative radiotherapy, and orthopaedic surgery. Both bone metastases and SREs are associated with unfavorable prognosis and greatly affect quality of life. Few epidemiological data exist on SREs after primary diagnosis of BrCa and subsequent bone metastasis. We therefore estimated the incidence of bone metastases and SREs in newly-diagnosed BrCa patients in Denmark from 1999 through 2007.

**Methods:**

We estimated the overall and annual incidence of bone metastases and SREs in newly-diagnosed breast cancer patients in Denmark from January 1, 1999 to December 31, 2007 using the Danish National Patient Registry (DNPR), which covers all Danish hospitals. We estimated the cumulative incidence of bone metastases and SREs and associated 95% confidence intervals (CI) using the Kaplan-Meier method.

**Results:**

Of the 35,912 BrCa patients, 178 (0.5%) presented with bone metastases at the time of primary breast cancer diagnosis, and of these, 77 (43.2%) developed an SRE during follow up. A total of 1,272 of 35,690 (3.6%) BrCa patients without bone metastases at diagnosis developed bone metastases during a median follow-up time of 3.4 years. Among these patients, 590 (46.4%) subsequently developed an SRE during a median follow-up time of 0.7 years. Incidence rates of bone metastases were highest the first year after the primary BrCa diagnosis, particularly among patients with advanced BrCa at diagnosis. Similarly, incidence rates of a first SRE was highest the first year after first diagnosis of a bone metastasis.

**Conclusions:**

The high incidence of SREs following the first year after first diagnosis of a bone metastasis underscores the need for early BrCa detection and research on effective treatments to delay the onset of SREs.

## Background

Breast cancer (BrCa) is one of the most commonly diagnosed cancers among women in the industrialized world [1], accounting for 28% of all new cancer cases in women in Denmark in 2008 [2]. At BrCa diagnosis, approximately 5%-6% of women present with distant spread [3,4], with bone representing the most common site of metastatic lesions. More than half of women, who present with metastatic breast cancer at primary diagnosis, will develop bone metastases [5]. Bone metastases in BrCa patients are dominated by osteolytic lesions, which increase the risk for skeletal-related events (SREs), defined as pathological fractures, spinal cord compression, bone pain requiring palliative radiotherapy, and orthopaedic surgery [6].

Published data on incidence rates of bone metastases and SREs after primary diagnosis of BrCa and subsequent bone metastasis are few. One Canadian study evaluated the pattern of metastastic disease in 180 patients with triple-negative (*i.e.*, estrogen receptor-negative, progesterone receptor-negative and HER2/neu-negative) BrCa compared with other subgroups of BrCa patients (N = 1,428). The risk of developing bone metastases within 10 years after diagnosis was 7%-9% for all subgroups [7]. Hortobagyi *et al. *evaluated the efficacy of bisphosphonates in reducing skeletal complications in patients with BrCa and bone metastases in a clinical trial setting [8,9]. They found that the median time to the first SRE was 13.9 months among bisphosphonate-treated women and 7.0 months in the placebo group (P = 0.001) [9].

The need remains for general population data on the incidence of bone metastases and SREs among unselected BrCa patients. Such data would allow further understanding of the clinical course of BrCa and related health care demand. We therefore estimated the incidence of bone metastases and SREs using a large population-based cohort of newly-diagnosed BrCA patients in Denmark from 1990 to 2007.

## Methods

We conducted this population-based cohort study in Denmark (population ~ 5.4 million inhabitants). The entire population receives tax-supported health care from the Danish National Health Service, with free access to hospital care. All BrCa patients receive care in specialised oncology centres within public hospitals operating under the auspices of the Danish National Health Service. Since 1968, the Danish Civil Registration System has kept up-to-date electronic records on date of birth, gender, change of address, date of emigration, and changes in vital status for all Danish residents.^10 ^From the Central Office of Civil Registration, each resident in Denmark is assigned a unique 10-digit civil registration number, which allows unambiguous linkage among all of Denmark's population-based registries [10].

### Breast cancer patients

We identified all patients in the Danish National Patient Registry (DNPR) with a first primary diagnosis of BrCa (*i.e.*, recurrent or relapsed cases were not included) recorded between January 1, 1999 and December 31, 2007 (during this period, there was no formal mammography screening program in Denmark). The DNPR collects electronic data on inpatient, outpatient and emergency room visits. For each hospitalization, DNPR files include dates of admission and discharge, any surgical procedure performed, and up to 20 discharge diagnoses. Since 1994, information has been coded according to the *International Classification of Diseases*, 10^th ^revision (ICD-10) [11]. BrCa patients were identified using ICD-10 code C50.x. Code C79.5 was used to identify bone metastases. We included both inpatient and outpatient diagnoses.

### Stage at diagnosis

Information on stage at BrCa diagnosis was obtained from the Danish Cancer Registry (DCR) until December 31, 2007. The extent of spread of the tumour at the time of diagnosis was recorded as local, regional, or distant metastases (*i.e*., summary staging) in the DCR. From January 1, 2004 information on stage was recorded according to the "TNM Classification of Malignant Tumors" cancer staging system. Conversion of the TNM classification system to the summary staging system is presented in Table 1.

**Table 1:** Translation of AJCC groupings into summary staging for breast cancer.

Stage	TNM categories
Localized	T1-4, N0, M0
Regional	T1-4, N1-3, M0
Distant	T1-4, N1-3, M1
0	Tis, N0, M0
I	T1, N0, M0
IIA	T0, N1, M0
	T1, N1, M0
	T2, N0, M0
IIB	T2, N1, M0
	T3, N0, M0
IIIA	T0, N2, M0
	T1, N2, M0
	T2, N2, M0
	T3, N1, M0
	T3, N2, M0
IIIB	T4, N0, M0
	T4, N1, M0
	T4, N2, M0
IIIC	Any T, N3, M0
IV	Any T, Any N, M1

### Skeletal-related events

The DNPR was used to identify SREs after BrCa diagnosis, with surgical procedures coded according to the Danish version of the Nordic Classification of Surgical Procedures (NCSP). For each patient, we recorded the following SREs: 1) radiation to the bone (NCSP-code: BWGC1 in addition to a bone metastasis diagnosis), 2) pathological or osteoporotic fractures in addition to a bone metastasis diagnosis (see Table 2 for codes), 3) bone surgery (NCSP-code: KNAGxx in addition to a bone metastasis diagnosis), and 4) spinal cord compression in addition to a bone metastasis diagnosis (see Table 2 for codes).

**Table 2:** Translation of fracture categories into discharge diagnoses in ICD-10.

Fracture category	**ICD-10 codes**:
Fractures of the vertebrae, ribs and pelvis, femur and distal forearm	M80.0, M84.4, M90.7, S12.0-12.9, S22.0, S22.1, S32.0-S32.8, S72.0-S72.9, S52.5-S.52.6
Spinal cord compression	M43.9, M48.5, M54.5, M54.6, M54.9, G95.2, G95.8

### Follow up

Patients were followed from the date of their BrCa diagnosis until the development of bone metastases or/and SREs, emigration, death, or April 1, 2008, whichever came first.

### Statistical analysis

We computed the cumulative incidence (%) and incidence rates of bone metastases during follow up among patients diagnosed with BrCa, treating death as a competing risk [12], and plotted these estimates as a function of time since BrCa diagnosis. Similarly, we calculated the cumulative incidence and incidence rates of SREs among BrCa patients diagnosed with bone metastases and plotted these estimates as a function of time since bone metastasis diagnosis.

To evaluate age at BrCa diagnosis as a risk factor for bone metastases, we stratified the analyses according to the following age groups: =60 years, 60-69 years, and 70+ years. We also stratified incidence of bone metastases and SREs by summary stage at BrCa diagnosis in order to evaluate the impact of BrCa stage on these outcomes.

This study was approved by the Danish Protection Agency (Record no. 2006-41-6387). The statistical software SAS, version 9.2 (SAS Institute Inc., Cary, NC), was used for all statistical analyses.

## Results

### Patient demographic characteristics

**Figure 1:**
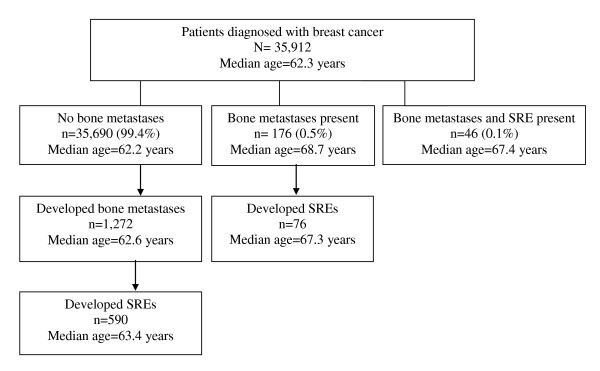
**Flowchart of the breast cancer study cohort (N = 35,912), according to presence of bone metastases and subsequent occurrence of skeletal-related events (SREs): Denmark, 1999-2007**.

We identified 35,912 patients with a first primary BrCa in Denmark from 1999 through 2007. The median length of follow up was 3.5 years (range, 0-9.2 years). The median age at BrCa diagnosis was 62.3 years (range, 18-104 years). Among those who developed bone metastases, the median age at BrCa diagnosis was 62.2 years (range, 25-95 years), and among those with bone metastases who developed an SRE, the median age at BrCa diagnosis was 67.4 years (range, 25-95 years). A flowchart of the BrCa study cohort according to presence of bone metastases and subsequent occurrence of SREs is presented in Figure 1. In the subset of BrCa patients (1999 through 2006) for whom we had information on primary stage of BrCa at diagnosis (n = 31,761), 13,515 (43%) patients presented with localized disease, 12,452 (39%) presented with regional disease, 1,557 (5%) presented with metastases, and 4,237 (13%) patients had missing stage data (data not shown).

The distribution of first-recorded SRE type among BrCa patients who developed an SRE (n = 712 patients) during the follow-up period was as follows: 394 (55%) had radiation to the bone, 133 (19%) had a pathological or osteoporotic fracture, 42 (6%) had bone surgery, and 143 (20%) had spinal cord compression as their first-recorded SRE type.

### Incidence of bone metastases and SREs

**Figure 2:**
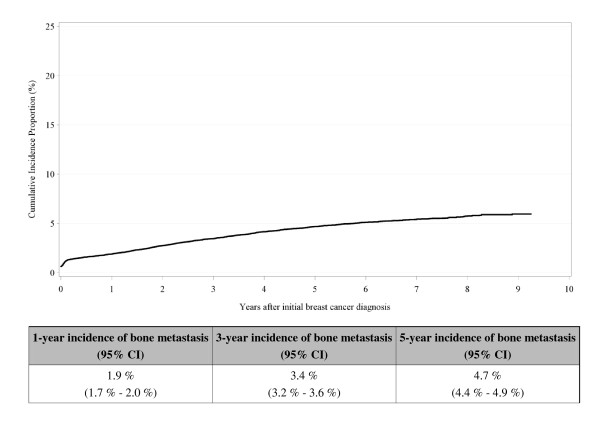
**Cumulative incidence of bone metastases among breast cancer patients (N = 35,912), Denmark, 1999-2007**.

**Figure 3:**
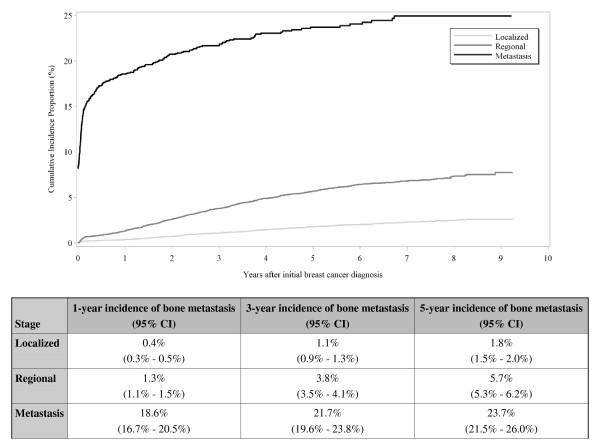
**Cumulative incidence of bone metastases among breast cancer patients (N = 35,912) by stage of disease at diagnosis, Denmark, 1999-2007**.

**Figure 4:**
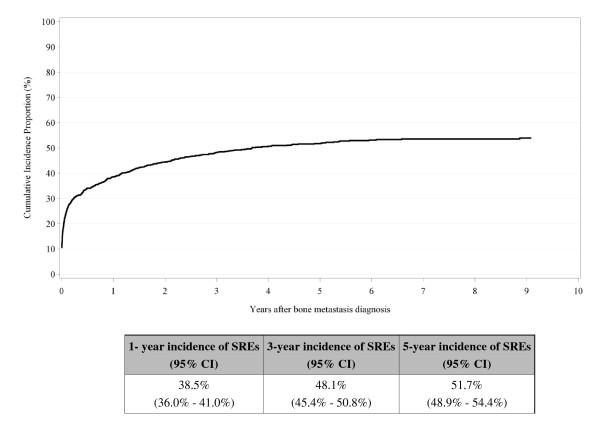
**Cumulative incidence of skeletal-related events among breast cancer patients with bone metastases (N = 1,272), Denmark, 1999-2007**.

Figure 2 shows the cumulative incidence of bone metastases among BrCa patients during follow up. The steepest increase appears in the first year after the primary diagnosis of BrCa [1-year cumulative incidence = 1.9% (95% CI: 1.7%-2.0%)], with the highest incidence observed among patients with an advanced stage at primary diagnosis (Figure 3). Figure 4 shows the cumulative incidence of SREs among BrCa patients with bone metastases during follow up. The steepest increase in SREs is seen the first year after bone metastasis diagnosis [1-year cumulative incidence = 38.5% (95% CI: 36.0-41.0%)], with no difference by primary stage at BrCa diagnosis (data not shown). We found no difference in incidence of bone metastases and SREs by age at BrCa diagnosis (data not shown).

Table 3 presents the incidence rates of bone metastases among all BrCa patients, overall and by stage, from the first year following primary BrCa diagnosis up to five years afterwards. It also presents the incidence rates of SREs among BrCa patients with bone metastases from the first year following primary diagnosis of bone metastases up to five years afterwards. A total of 1,494 (4% of 35,912) BrCa patients were diagnosed with bone metastases either at the time of BrCa diagnosis or during follow up. The incidence rate of bone metastases was highest the first year after the primary diagnosis of BrCa [incidence rate (IR) = 13.6 (95% CI: 12.4-14.9)] per 1,000 person-years (PY). This rate declined to 8.8 (95% CI: 8.2-9.5) per 1,000 PY from the second to the fifth year after the primary BrCa diagnosis. A total of 712 (47.6% of 1,494) BrCa patients with bone metastases were diagnosed with an SRE either at the time of primary BrCa diagnosis or during follow up. Similar to the incidence of bone metastases after the primary BrCa diagnosis, the incidence rate of SREs was highest the first year after the primary diagnosis of bone metastases [IR = 759.2 (95% CI: 662.0-870.5) per 1,000 PY]. This rate declined to 513.8 (95% CI: 465.4-567.3) per 1,000 PY from the second to the fifth year after diagnosis of bone metastases.

**Table 3:** Incidence rates of bone metastases among breast cancer patients, and incidence rates of skeletal-related events (SREs) among breast cancer patients with bone metastases, from the first year after primary diagnosis of breast cancer and bone metastases, respectively, up to five years after diagnosis, Denmark, 1999-2007.

	Event	n	Year 1	Year 2 - Year 5	Year 1 - Year 5
			**Per 1,000 person-years (95% CI)**		
			
All patients	Bone metastasis	1,272	13.6 (12.4 - 14.9)	8.8 (8.2 - 9.5)	10.2 (9.6 - 10.8)
	First SRE	712	759.2 (662.0 - 870.5)	513.8 (465.4 - 567.3)	578.0 (533.4 - 626.2)
Localized	Bone metastasis	237	3.3 (2.5 - 4.4)	3.9 (3.3 - 4.5)	3.7 (3.2 - 4.3)
	First SRE	117	503.1 (285.7 - 885.9)	574.7 (462.8 - 713.5)	564.4 (461.1 - 690.9)
Regional	Bone metastasis	663	12.9 (11.1 - 15.1)	13.5 (12.3 - 14.8)	13.3 (12.3 - 14.5)
	First SRE	310	774.6 (596.1 - 1006.5)	560.7 (490.7 - 640.6)	594.5 (527.8 - 669.5)
Distant	Bone metastasis	358	172.5 (147.9 - 201.4)	49.7 (38.8 - 63.6)	101.8 (89.3 - 116.0)
	First SRE	176	760.1 (620.9 - 930.4)	421.5 (329.9 - 538.5)	573.5 (490.7 - 670.2)

The incidence of bone metastases was higher after the first year if the BrCa patient was diagnosed at a more advanced stage: localized spread: IR = 3.3 (95% CI: 2.5-4.4) per 1,000 PY; regional spread: IR = 12.9 (95% CI: 11.1-15.1) per 1,000 PY, and metastatic spread: IR = 172.5 (95% CI: 147.9-201.4) per 1,000 PY (Table 3). However, there were no differences in the rates of SREs subsequent to bone metastases by stage of disease at BrCa diagnosis (Table 3).

## Discussion

In this large population-based study conducted within a well-defined Northern European population, we found the five-year incidence rate of bone metastases among BrCa patients to be 10 per 1,000 PY; the corresponding incidence rate of SREs was 578 per 1,000 PY. The incidence rate of bone metastases was highest the first year after primary diagnosis of BrCa and higher if the BrCa patient was diagnosed at a more advanced stage. The incidence rate of SREs was highest the first year after diagnosis of bone metastases and showed no differences across stage at BrCa diagnosis.

The lack of a formal mammography screening program in Denmark during the study period may explain the relatively high prevalence of BrCa patients with an advanced stage of BrCa at primary diagnosis. Since population-based mammography screening is an accurate tool for early BrCa detection [13], we would expect incidence rates of bone metastases to be lower in countries offering this screening. This was the case in a German observational study that evaluated BrCa incidence rates and tumor characteristics (*i.e*., the distribution of invasive tumor size) before and after implementation of mammography screening [13]. The incidence rate of BrCa before implementation of the screening programme was 297.9 per 100,000 person-years. During the implementation of screening, this rate rose to 532.9 per 100,000 person-years. Of the 349 cancers detected with screening, 76% were invasive, compared to 90% of cases not detected with screening during the same period. Furthermore, there was a difference in nodal status between cancers detected with and without the screening program [13].

Breast cancer patients have predominantly osteolytic bone metastases, which is characterised by increased bone degradation resulting from enhanced osteoclastic activity [14]. This activity in the bone causes pain, which is consistent with the most frequent SRE being 'palliative radiation to the bone' in our study. Bisphosphonates are potent inhibitors of osteoclast-mediated bone resorption [15]; accordingly it has been shown that use of bisphosphonates delay the time to an SRE among BrCa patients [9,16]. Data on bisphosphonate use has been recorded in the DNPR since 2002; however, the completeness of this registration is unknown. In our cohort of 35,912 breast cancer patients, only 1,090 (3%) had a record of bisphosphonate use prior to a recorded bone metastasis diagnosis, and an additional 245 women had a record of bisphosphonate use subsequent to a recorded bone metastasis diagnosis. Because the quality of data on bisphosphonate use is unknown in the DNPR, we did not include these data in our study.

The study's population-based cohort design enabled us to identify all hospital discharge and outpatient diagnoses of BrCa, bone metastases, and SREs over an 8-year period, thereby minimizing the risk of referral and diagnostic bias. However, the validity of our findings depends on the accuracy of the coding of these events and completeness of reporting in the DNPR. In a previous study, we found that bone metastases and SREs secondary to BrCa were underestimated in the DNPR. Completeness of DNPR recording of bone metastases was 32%, and the positive predictive value was 86% compared with a medical record review. This may explain why the risk of bone metastases among BrCa patients in our study was lower than the one reported among BrCa patients in a previous study (4% vs. 9%, respectively)[7]. Completeness of DNPR recording of SREs was 75%, and the positive predictive value was 75%, compared with a medical record review [17]. Given that we have a higher completeness of reporting of bone metastases and SREs compared to bone metastases alone, we may have overestimated the risk of SREs in patients with bone metastases (i.e., bone metastases in combination with an SRE is more likely to be identified than bone metastases and no SRE). In addition, we coded pathological and osteoporotic fractures under one code. This most likely overestimated the incidence of SREs among breast cancer patients because some of the fractures might have been osteoporotic. However, we do think that when a breast cancer patient is diagnosed with a bone metastasis and subsequently develops a fracture, it is most likely pathological. Thus, we anticipate a high proportion of fractures in this category of SREs as pathological.

There are several potential explanations for the degree of under-coding of bone metastases. Diagnostic procedures used to screen for bone metastases in breast cancer patients may depend on the patient's expected prognosis. For instance, if a patient's overall status is deemed inappropriate for radiation therapy or surgery (i.e., poor prognosis) then there may be little incentive to code bone metastases. Additionally, the numeric ICD-10 coding system is used to characterize obvious medical events; however, a bone metastasis may not be clinically obvious. Finally, reporting of bone metastases are not mandatory in Denmark, which may decrease the tendency for physicians and specialists to code these events.

## Conclusions

In conclusion, we found a high incidence rate of SREs among BrCa patients with bone metastases, particularly during the first year following a bone metastasis diagnosis. This highlights the need for early detection of BrCa and use of existing effective treatments that can delay the onset of SREs.

## Competing interests

This study was supported in part by Amgen Inc, USA. Mellissa Yong is currently employed and has stock ownership in Amgen Inc. Jon Fryzek has stock ownership in Amgen Inc and, at the time this study was conducted, was an employee of Amgen Inc.

## Authors' contributions

AJ contributed to the conception and design, administrative support, provision of study material, data analysis and interpretation, and manuscript writing. JJ provided analytic support and interpretation and manuscript writing. MN contributed to the conception and design, data analysis and interpretation, and manuscript writing. MY contributed to data analysis and interpretation and manuscript writing. JF contributed to the conception and design, data analysis and interpretation, and manuscript writing. HS contributed to the conception and design, financial and administrative support, provision of study material, collection and assembly of data, and data analysis and interpretation. All authors read and approved the final manuscript.

## Pre-publication history

The pre-publication history for this paper can be accessed here:

http://www.biomedcentral.com/1471-2407/11/29/prepub
